# From potential to podium: what we still don't know about talent identification and development in Para athletics, a scoping review

**DOI:** 10.3389/fspor.2026.1782132

**Published:** 2026-06-15

**Authors:** Annmarie Carroll, Alison O’Riordan, Kate Pumpa

**Affiliations:** 1 School of Public Health, Physiotherapy, and Sport Science, University College Dublin, Dublin, Ireland; 2 Paralympics Ireland, Irish Sport HQ, Blanchardstown, Dublin, Ireland; 3 Institute for Sport and Health, University College Dublin, Dublin, Ireland; 4 Sport and Exercise Medicine, Queen Mary University of London, London, United Kingdom

**Keywords:** athlete development, Para athletics, Para track and field, talent development, talent identification

## Abstract

The ability to identify and develop talented athletes effectively can increase the likelihood of sporting success and thus increase returns on invested resources for stakeholders. Talent identification and development (TIdD) is well studied in sports which are highly monetised, such as soccer and rugby, however, it is less researched in Para sport. The purpose of this scoping review is to synthesise and map the current landscape of research on TIdD in Para athletics (PA) to allow for evidence-based recommendations to be made for applied practice. This study followed a five-step framework for conducting scoping reviews and searched five major databases [PubMed, SPORTDiscus, PsycINFO (via EBSCO), ERIC (via ProQuest), and SCOPUS (via Elsevier)], reference lists and existing networks, relevant organisations and conferences, for research pertaining to TIdD in PA. The search returned 1,786 results, of which, nine papers were included in this review. Of the nine publications related to TIdD in PA, five were published within the last 5 years, and four reported on physical testing data. The most investigated track and field discipline was sprinting and there was a notable absence of representation for several classifications. The review found that TIdD practices in PA are primarily centered on physical assessments and the analysis of demographic data, including birthdates and impairment-related factors. Further research is needed in this area, with research teams encouraged to give greater attention to diversity and critically reflect on the broader applicability of their findings.

## 1 Introduction

The purpose of talent identification and development (TIdD) is to recognise those amateur, early career athletes with potential to progress to elite level, and to provide them with the necessary resources to increase their chances of achieving sporting success (1–3). Effective TIdD can ensure the correct allocation of resources and increased likelihood of return on investments (4). While TIdD processes are well-established in non-disabled sport, the same processes have only recently gained traction in Para sport (5, 6). This may be a consequence of the Paralympic movement, of which athletics is the biggest sport in terms of participation of athletes and countries (7). As the competitiveness of Para athletics (PA) increases (8), so too does the necessity for effective TIdD. Identifying talented athletes, and investing in them earlier, may equate to improved sporting success.

Compared to non-disabled sport, Para sport has added complexities, such as differences in developmental trajectories related to the nature of an athlete's impairment, including the onset, severity and classification (9). Classification is directly associated with the athlete's impairment and aims to group athletes together based on how their impairments impact their ability to perform their event, allowing for fairer competition (10). Within Para sport, classification functions as a foundational structural mechanism that organises eligibility and competition while shaping athlete pathways and distributive outcomes across the Paralympic system. Classification defines who is permitted to compete and how athletes are grouped into sport classes to minimise the impact of impairment on performance (11–13). Classification is underpinned by an evolving evidence based rationale with the International Paralympic Committee (IPC) Position Stand mandating that classification systems be built upon empirical evidence linking impairment and performance in each sport (14). An example of this evolving evidence base and how it can impact an athlete's development and performance pathway is the introduction of new classification testing in 2018 which impacted athletes in sports classes T/F 20 (intellectual impairments), T/F 31–34 (co-ordination impairments, competing in a seated position) and T/F 35–38 (co-ordinations impairments), and included the introduction of new sport classes T61–64 (athletes who run or jump with prosthetic lower limbs) (15). As such, classification must be understood not merely as an administrative procedure or a description of functional ability, but as an organising principle that shapes athlete experiences, constrains and enables performance trajectories, and can reinforce or challenge existing inequalities within Para sport. For these reasons, this review adopts a performance system lens to allow the interpretation of the findings to reflect classification as a system-level organisational principle.

Alongside these, the broader biopsychosocial elements associated with physical disabilities, like resilience, sport-specific skill acquisition, and life commitments, must be considered (16). Additionally, Para sport is associated with smaller resource pools (17). For instance, in Ireland, the Irish Sports Monitor (18) reported that the gap between the number of non-disabled people and people living with disabilities participating in sports is widening. These nuances may make Para sport research more challenging and may deter some researchers. This lack of consideration for Para sport may be contributing to the adoption of non-disabled sports practices, policies and pathways, including those related to TIdD, to Para sport settings (19).

Youth athlete development pathways in general are well-documented (20, 21) and have been conceptualised through frameworks such as the Long-Term Athlete Development (LTAD) model (22) and the Foundations, Talent, Elite, Mastery (FTEM) framework (23). However, these models largely overlook the unique considerations inherent to Para sport, highlighting the need for tailored approaches that reflect the distinct experiences and developmental trajectories of Para athletes, including specifications for acquired and congenital impairments. From the research that has been conducted, key elements of successful Para athlete development include early sport-related experiences within family contexts, engagement in multiple sports during childhood (24), and the adoption of a holistic developmental approach (25).

Due to Para sport research requiring context specificity, i.e., classification, and the limited literature specifically focused on PA, a scoping review was considered the most appropriate method for synthesising the existing evidence. This approach enabled the research team to systematically map the current body of work and highlight gaps in knowledge within the field. Therefore, this review aims to map the current literature pertaining to TIdD practices in PA.

## 2 Methods

### 2.1 Design and search strategy

The guidelines of the Arksey and O'Malley's 2005 framework were followed. According to these guidelines the scoping review consists of five steps: (1) identifying the research question; (2) identifying relevant studies; (3) study selection; (4) charting the data; (5) collating, summarising and reporting the results. The search was performed across several electronic databases, reference lists and existing networks, relevant organisations and conferences.

The protocol for this study was drafted using the Preferred Reporting Items for Systematic Reviews and Meta-analysis for Scoping Reviews (PRISMA-ScR) and the Joanna Briggs Institute (JBI) Populations, Concept, Context (PCC) framework (26). This review aimed to answer the question: what are the current talent identification and development practices in Para athletics? To identify potentially relevant sources of evidence a search of five databases [PubMed; SPORTDiscus; PsycINFO (via EBSCO); ERIC (via ProQuest); SCOPUS (via Elsevier)] was performed.

A search strategy was developed to identify all relevant studies related to TIdD in PA. In this review, a Para athlete is defined as an athlete who is living with an eligible impairment (as defined by the IPC) and is thus, through the process of classification, deemed eligible to compete at World Para Athletics approved events (27). Searches were conducted in English; however, peer-reviewed articles published in any language were considered for inclusion. Studies from 1960 (the year of the inaugural Paralympic Games) to the date of the search (28th May 2025) were eligible for inclusion. Eligible studies were required to encompass a PA (i.e., Para track and field) population of any age or gender, and include the concepts of either athlete identification or development of athletes (e.g., through development pathways or training interventions). All study designs were eligible for inclusion. An experienced librarian was consulted to assist with developing keywords and search terms (see Supplementary Material S1 for the full search string). Wildcards, truncations and common misspellings of Paralympian/Paralympic/Para athletic/Para athlete were employed to maximise results. Grey literature was included in the search. For this reason, the above databases and Google Scholar were searched to capture any current ongoing research relevant to the research question. The final protocol was registered prospectively with Open Science Framework on 6th August 2024 (https://osf.io/43jqc).

### 2.2 Study selection

The search findings were uploaded to Covidence for the purpose of screening. Title and abstract screening were performed independently by two research team members who also performed the full text screening using the same inclusion and exclusion criteria (see Supplementary Material S2 for the inclusion and exclusion criteria). A third researcher was available to resolve any conflicts. To ensure due diligence, backward and forward citation tracking of the included studies was performed. Hand searching was the final level of searching conducted. In those studies where the participant pool was undefined, i.e., the study was related to Para sport and it was unclear if the data was representative of stakeholders in PA, contact was made with the corresponding authors for clarification. A high response rate from the corresponding authors meant that no studies were excluded due to lack of clarification on the participant pool.

### 2.3 Study appraisal

Although quality assessment is not a necessity for a scoping review, due to the newness of the space and the need for definitive practical guidelines, the included studies were assessed using the Rosendal Scale (28). Previous scoping reviews have used the Rosendal scale for the appraisal of included studies as it allows for assessment of various factors which are associated with the minimisation of bias (29). Scoring of each publication was determined by dividing the number of “yes” responses by the number of relevant items, with excellent methodological quality indicated by a score of ≥60%.

### 2.4 Data extraction

Given the anticipated heterogeneity in study designs, population and outcome measures of this search, findings were synthesised using a descriptive and thematic approach. Studies were not excluded based on methodological differences; rather, variation across studies was retained to allow for a comprehensive mapping of the evidence base. This approach enabled the identification of patterns and differences across contexts, participant groups, and study characteristics. Article characteristics (e.g., country of origin, study design) and contextual factors (e.g., participant demographics, impairment type) were extracted, recognising these as structural components of national Para sport performance systems. Country of origin was treated as indicative of national level system organisation and policy contexts and thus refers to the structures, organisations, policies, and environments that govern and support sport within a country. Impairment type was considered in relation to classification and eligibility structures that shape access to TIdD pathways. Studies were subsequently grouped according to recruitment and participant demographics, study design, geographical spread, and TIdD topics to map how different performance system configuration's structure athlete development opportunities.

## 3 Results

**Figure 1:**
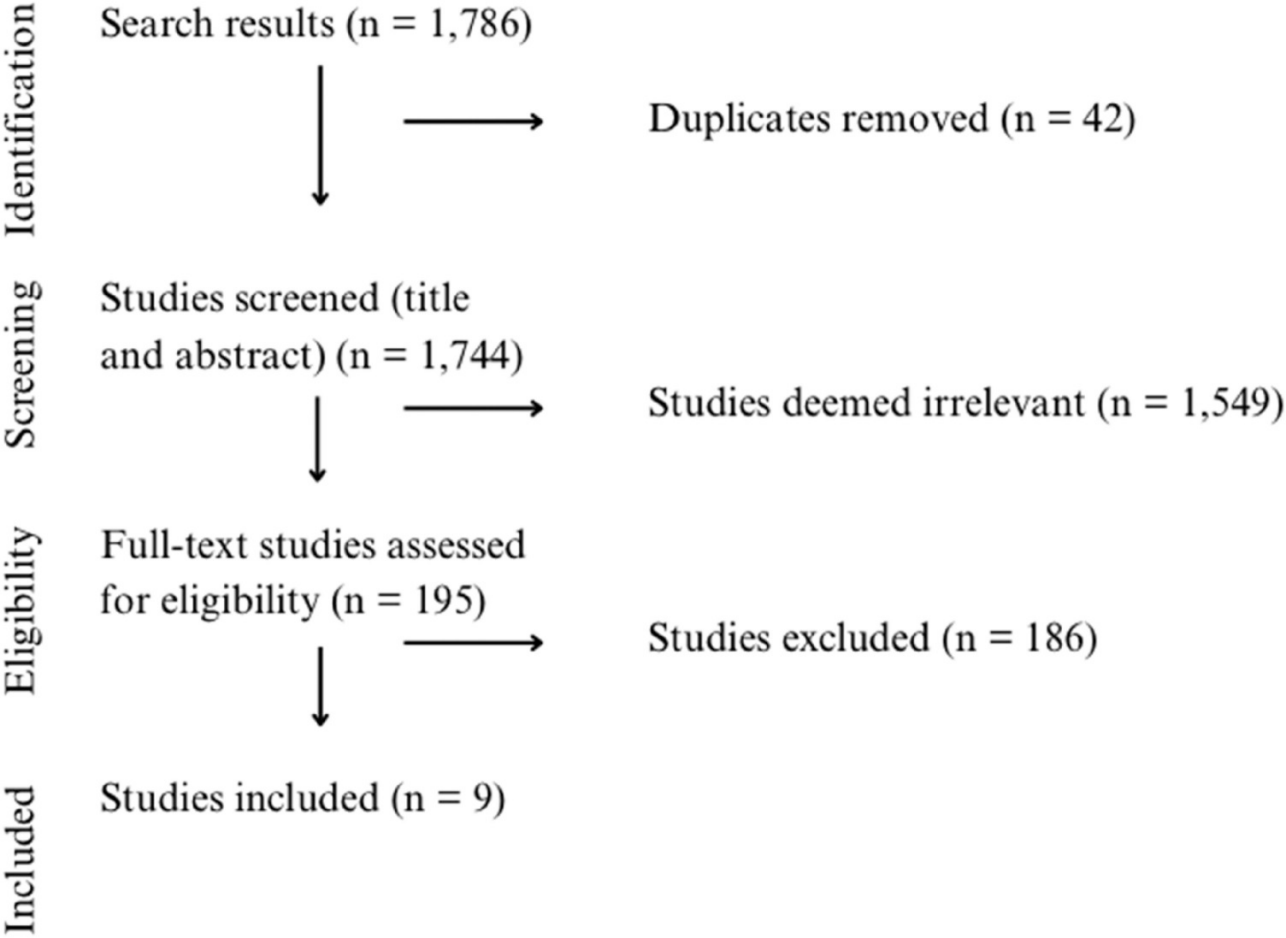
PRISMA flow chart.

The search returned 1,786 studies for title and abstract screening, after the removal of duplicates, title and abstract screening was performed on 1,744 studies. Full text reviews were performed on 195 studies with nine studies included for analysis (see Figure 1). Publication dates range from 2011 to 2025. The characteristics of the nine included studies are presented in Table 1, which outlines key details such as the athletics event(s) and impairment type(s) examined in each study, along with a summary of the main findings.

**Table 1:** Study Characteristics.

**Title**	**Overall Theme**	**Other themes**	**Study Methodology**	**Participants / Data points**	**Aspect of TID**	**Athletics Specific**	**Athletics event included in analysis**	**Eligible impairments included in analysis**	**Study findings**
Andrews BS; Ferreira S; Bressan ES**,** 2011	Biomechanics	Sprint Performance, Biomechanics, Comparison to ND, Quantitative, Athlete development, TIdD tool	Descriptive quantitative	n = 6 (all male) South African Paralympic athletes, aged 23-27	Talent development	Yes	Track	VI, Ataxia/Hypertonia/Athetosis, Limb difference	The sprint biomechanics of the Para athletes did not fit into the norms of ND sprinters, but analysis of sprint starts was effective for identifying areas for improvement and were relevant for coaching practices.
Loturco I; Winckler C; Kobal R; Cal Abad CC; Kitamura K; Veríssimo A W; Pereira LA; Nakamura FY**,** 2015	Performance prediction	Quantitative, Classification, Sprint Performance, Effects of impairments, Physical testing	Quantitative comparison	n = 15 (7 male, 8 female) Brazilian Paralympic sprinters with VI	Talent development	Yes	Track	VI	Loaded and unloaded jump tests can be used for predicting sprint performances in the 100m and 200m dash in Paralympic VI sprinters. The data from the jump tests can be used in a multiple linear regression equation to predict times.
Connick MJ; Beckman EM; Spathis JG; Deuble R; Tweedy SM, 2015	Classification	TIdD tool, Physical testing, ROM, Coordination, Quantitative	Cross sectional	n = 41 (all male) runners, aged 24.3±9.4 (RBI) and 23.1±4.1 (NDR). RBI = 13, NDR = 28. T35 (n = 2); T36 (n = 1); T37 (n=6); and T38 (n =4)	Classification	Yes	Track	Ataxia/Hypertonia/Athetosis, TBI/Stroke	Five of the ROM measures significantly affected sprint performance in RBI and were deemed valid for the purposes of classifying impairments in classes T35–T38.
Beckman EM; Connick MJ; Tweedy SM, 2016	Classification	RAE, Quantitative, Sporting success (factors of)	Cross sectional	n = 41 (all male) runners, aged 24.3±9.4 (RBI) and 23.1±4.1 (NDR). RBI = 13, NDR = 28. T35 (n = 2); T36 (n = 1); T37 (n=6); and T38 (n =4)	Classification	Yes	Track	Ataxia/Hypertonia/Athetosis, TBI/Stroke	The measures investigated (leg flexion, extension and plantarflexion) were significantly different in RBI compared with NDR indicating the tests were able to capture strength impairments in this population.
Bayarslan B; Özsoy D; Çevik A**,** 2023	RAE	RAE, Quantitative, TIdD tool	Basic qualitative research	n = 169 (102 male, 67 female) Paralympic Turkish athletes from 15 different sports (PA n = 20)	Talent identification	No	Not specific	Not relevant	Of the 20 birthdates included for PA, 6 were from the first 6 months of the year while 14 were in the last 6 months.
Teymouri M; Daneshmandi H; Majelan AS**,** 2023	Talent Identification	TI tool, Quantitative	Descriptive survey design	n = 98 (52 male, 46 female) Iranian seated throwers	Talent identification	Yes	Field	Not specified	All T-values in CFA were statistically significant (T > 1.96), indicating strong construct validity. Participants rated most test items (fitness, anthropometric) above the desirable level, supporting the content validity and relevance of the questionnaire.
Peake R; Davies LE, 2024	Sports policy	Classification, Sprint Performance, ROM, Coordination, Physical testing, Quantitative	Mixed methods approach	n = 42 GB athletes, n = 38 coaches, n = 7 interviewed athletes, n = 5 interviewed coaches, n = 3 interviewed UK support staff	Talent development	Yes	Track, Field	VI, Lower limb impairment, Ataxia/Hypertonia/Athetosis	The is some correlation between factors that influence sporting success in ND sports and Para sport. There is variance in considerations between acquired and congenital impairments and across event groups (i.e., throws, jumps, sprints and middle-long distance running).
Solon-Júnior LJ; Albuquerque Melo T; Oliveira JK; Silva Neto LV; Teixeira Barbosa B; de Oliveira Castro H; de Sousa Fortes L**,** 2024	RAE	Performance predictions, Physical testing, Quantitative, RAE	Descriptive, cross-sectional, observational	n = 700 birth dates (all male) from Brazilian athletes, aged U16, U18, U20	Talent identification	Yes	Track, Field	Not specified	The RAE has an influential role in track and field Para athletes, but it does not seem to be related to performance.
Severin AC; Kinderen A; Baumgart JK**,** 2025	Impairment related effects	Effects of impairments, Performance predictions, Quantitative, TIdD tool	Retrospective data analysis	n = 4,331 data points from athletics; n = 1,638 PA athletes	Talent identification	No	Track, Field	VI, Ataxia/Hypertonia/Athetosis, Impaired MP, SCI, Impaired ROM, Lower limb impairment, II, Short stature	Athletes with a congenital impairment had a significantly higher likelihood of winning a medal compared to those with an acquired impairment in PA.

ND: non-disabled; VI: visual impairments; ROM: range of motion; RBI: runners with brain injury; NDR: non-disabled runners; TBI: traumatic brain injury; RAE: relative age effect; PA: Para athletics; CFA: confirmatory factor analysis; GB: Great Britain; UK: United Kingdom; MP: muscle power; SCI: spinal cord injury; II: intellectual impairment.

### 3.1 Recruitment and participants

Six studies recruited participants, with sample sizes ranging from 6 to 98 and ages between 15 and 36 years. Three studies used retrospective data: Solon-Júnior et al. (37) analysed birthdates of 700 PA athletes (U16, U18, U20); Bayarslan et al. (34) examined birthdates of 20 PA athletes; and Severin et al. (38) assessed 4,331 competition results from 1,638 PA athletes. Eight of the nine studies included data from males (30–33, 35, 37, 38). Four studies included data from females (31, 34, 35, 38). None of the studies recruited females only.

The studies varied in terms of the type(s) of impairments investigated. Three studies focused on specific impairments [visual = 1 (31); co-ordination impairments = 2 (32, 33)]. The remaining studies either included a multitude of impairments or did not refer to impairment type in their analyses. Seven of the nine studies are specific to PA, the remaining two (34, 38) investigate multiple sports but have findings which are specific to PA.

### 3.2 Study design

The methodologies employed across the studies varied widely, ranging from secondary data analysis to performance prediction models in Para sport. Four studies utilised quantitative physical testing to examine performance outcomes (30–33). Three studies reported on retrospective data analyses (34, 37, 38). One study employed a mixed-methods study design (36). One study implemented a standardised descriptive design to validate a questionnaire (35).

### 3.3 Geographical spread

The geographical spread of the studies, and thus the representation of national Para sporting systems, is broad. Australia and Brazil have published the majority of research in this space (*n* = 2 for both countries). There was one publication from each of the following countries: the United Kingdom, Turkey, Norway, South Africa and Iran.

### 3.4 Topics within talent identification and development in para athletics

Four of the studies focused on running performance (30–33). Correlations have been identified between jump tests, range of movement abilities and sprint performance (32, 33). One study focused on throwing performance (35). None of the studies related to jumping events. Two studies explored the relative age effect (RAE) (34, 37). One study addressed sport policy and programs, with an emphasis on factors attributable to success (36). One study investigated the effects of different impairment types on Paralympic medal success (38).

### 3.5 Assessment of reporting quality

Results of the quality assessment are presented in Supplementary Material S3. The average Rosendal score was 80 ± 7.5%, with all studies achieving a score rated as excellent (≥60%).

## 4 Discussion

This scoping review sought to map the current literature exploring TIdD in PA. The findings reveal a notable gap in research, characterised by limited diversity across both participant and data pools, which constrains the breadth of applicability. Furthermore, a scarcity of studies examining applied practice was evident. Researchers examining Para sport at a systems level reported the pathways through which Para athletes' access and progress within high-performance sport are shaped by a complex set of organisational, policy, and contextual factors that vary considerably across national systems (39, 40). The current findings extend this literature by moving beyond policy-level analysis to examine how TIdD processes operate in practice, reflecting calls within the field for greater attention to the structural conditions that either support or constrain Para athlete development (39). Viewed through a performance systems lens, these patterns reflect the structural configuration of Para sport systems, in which classification, funding priorities, and organizational capacity jointly determine what athletes get identified, supported, and ultimately studied. Collectively, the evidence suggests that improving TIdD in PA may require system-level approaches that address structural, organisational, and resource-related factors rather than focusing solely on individual athlete development. For example, classification processes and resource allocation strategies can jointly shape talent development (TD) pathways. Delays or uncertainty in classification may restrict access to competition, while system-level prioritisation of “medal-dense” classes can concentrate resources within specific groups. Together, these structural factors influence which athletes are identified, supported, and progressed, demonstrating that improvements in TIdD require coordinated system-level approaches rather than a sole focus on individual performance.

As expected, the research conducted to date has a distinctive cohort, for example, track athletes with visual impairments (VI) (classifications T11–T13), which indicates the findings may only be applicable to athletes competing within those specific classifications. Further, four studies included data on males only therefore, the findings may not be transferable to female populations. Geographical specificity, which is directly linked with national level sporting system contexts, is another factor that can influence the applicability of a study's findings due to demographics, resource availability and differences in data reporting. Although these patterns reflect the literature in this space to date, they also reflect the system-level mechanisms which produce these patterns. For example, the male-dominated participant samples likely reflect not only researcher bias but the historical bias towards males in sporting contexts that are a direct result of national system governance where support, media attention and sport entry points have favoured males.

This review sought to include TIdD research from all PA events and sports classes. However, most of the studies (four of nine) relate to sprint performance, with only one paper exploring TIdD in throws, and none exploring jump events. The higher volume of publications focused on track research may reflect the higher volume of track events, and thus medal opportunities, than field events: Paris 2024, saw 86 track events held, compared with 78 field events. Alternatively, this research bias may be explained by some classes being easier to study or better resourced. For example., in Iran, the research team of Teymouri et al. (35) investigated TIdD in seated throws., which includes athletes with classifications F31–34 (co-ordination impairments) or F51–57 (athletes with limb deficiency, leg length difference, impaired muscle power or impaired range of movement). At the most recent World Para Athletics Championships (New Delhi, 2025), Iran was represented by 19 athletes, 18 of whom successfully competed in throwing events resulting in Iran placing third in the overall medal table. Sample size limitations were also noted by Peake and Davies (36), who highlighted that their sample was limited to athletes competing as part of team Great Britain at the time of the study, resulting in underrepresentation of some classifications, and Severin et al. (38), who detailed challenges relating to gathering data for their study due to the categorisation of impairment related data as being sensitive health data. These examples highlight how classification-driven eligibility shaped the inclusion and exclusion of the studies in this review. From a performance systems perspective, these findings point to uneven allocation of Para sport resources within national high-performance structures, suggesting that development pathways may not be equally accessible to all impairment types and thus, favour some Para athletes. Unequal resource allocation not only favours certain athletes and thus impairment types but also determines who and what impairments become visible to science.

The rise in female participation rate in Para sport is evidenced by the most recent edition of the Games (Paris 2024) showcasing a record proportion of female competitors (45%), an improvement from 42% in Tokyo 2020, and over double the number of female athletes that lined up at Sydney 2000. This increase is not reflected in the research as only four of the nine studies investigated female related data, and only one included gender as a factor in its analysis (34). Further, Solon-Júnior et al. (37) reported that their rationale for the exclusion of female data was that there was not enough data available for analysis. Although this may reflect the proportion of female athletes competing at Paralympic Games, it is in keeping with the historical lack of female related sport science data (41, 42) including talent related research (43). This disparity likely reflects historical patterns of resource allocation and pathway prioritisation within Para sport, whereby male athlete development has received greater structural investment and, consequently, greater research attention. As research activity often aligns with high-performance priorities, the underrepresentation of female athletes in the literature may both reflect and reinforce existing system-level inequalities. Future research should address this gap by increasing the inclusion of female participants across mixed-gender studies, as well as through the development of female-specific research. Participation among female Para athletes is expected to continue rising, driven in part by proactive initiatives from governing bodies such as World Para Athletics, including the introduction of a female-only Grand Prix competition in 2025. However, without parallel investment in research and development structures, increased participation alone may not translate into optimised TIdD pathways for female athletes. If this lack of female-focused research persists, there is a risk that the disconnect between academic research and applied practice within PA will continue to widen.

The nine studies included in this review utilised a range of research designs and analytical approaches. Factors specific to Para sport research may constrain the selection of suitable study designs, for example, athlete classification. Limiting research to one sports class reduces confounding variables and may be the reason a number of the studies investigate a specific impairment or sports class (30, 32, 33). Limited sample size can be likened to research in rare disease settings. Just as rare disease research contends with limited sample sizes and high phenotypic variability (44, 45), Para sport research must navigate functional diversity within sports classes, which can result in challenges regarding the generalisability of findings to a wide cohort. Alternatively, this could also reflect athlete availability and the limited pool of participants within a national Para sport system eligible to participate in research. These methodological patterns are not merely technical decisions made by research teams but reflect underlying structural features of Para sport, including classification frameworks, small and fragmented athlete populations, and the organisation of national pathways. As such, research design in this context is inherently shaped by system-level constraints, where the need to balance scientific rigour with limited and unevenly distributed athlete populations influences study scope and generalisability. When conducting research in the Para sport space, researchers are encouraged to provide clear justification for their chosen sample sizes and study inclusion criteria, thereby enhancing transparency and interpretability across the field. Greater alignment between research design and system characteristics may also support the development of more contextually relevant and transferable evidence to inform TIdD practice.

Geography, and thus sporting nation system, can also influence applicability of findings. Variations in funding availability, institutional support, and access to athletes across national systems influence the feasibility and design of studies. In some nations, limited financial resources and infrastructural constraints restrict opportunities for large-scale or longitudinal investigations. Peake and Davies (36) reported how their findings may only be applicable to the United Kingdom (where the research was conducted) due to the variances between the attention and resources allocated to Para sport across countries. Moreover, social attitudes toward disability and differing levels of inclusion in sport can affect both participation rates and research engagement. For example, the findings from Solon-Júnior et al. and Bayarslan et al., which related to the RAE in PA in their countries, Brazil and Turkey respectively, had similar findings yet one major difference between the two studies is the sample size: Solon-Júnior et al. (37), analysed the birthdates of 700 PA athletes, while Bayarslan and colleagues analsyed the birthdates of 20 (34). In terms of PA participation between the two countries, at the latest Paralympics Games in 2024, Brazil was represented by 71 PA athletes while Turkey had 16. For Turkey, the 2024 Games even marked their first ever gold in PA. Further, Severin et al., (38) reported the difference between impairment related data of Northern and Southern hemispheres as a significant limitation of their study. These examples highlight that research outputs are not produced in isolation but are shaped by system-level factors, including investment strategies, pathway design, and the maturity of Para sport programmes within each country. This results in a research landscape that mirrors global inequalities in Para sport development, where well-resourced systems are more likely to generate both athletes and evidence. Therefore, the dominance of the nations of Australia and Brazil highlight the maturity of the country's national performance systems and their level of research integration. Disparities in these factors contribute to an uneven global representation of Para sport disciplines and athlete classifications in the literature. This uneven distribution of evidence may, in turn, reinforce existing system-level advantages, as nations with greater research capacity are better positioned to develop evidence-informed TIdD practices. Consequently, greater international collaboration and resource sharing are needed to ensure that Para sport research reflects the diversity of contexts and national systems worldwide.

Despite a similar proportion of athletes with acquired and congenital impairments, the latter consistently show a greater likelihood of achieving medal success across all impairment types (38). Severin and colleagues were unable to determine if this reflects a genuine performance advantage, or simply greater access to more medal opportunities for athletes with congenital impairments (38). At a performance systems level, this distinction may shape how athletes are prioritised within TIdD programmes and how resources are allocated across impairment groups. Although the studies in this review did not directly explore these structural dynamics, the results highlight the need to view classification not only as an athlete characteristic but also as a system-level mechanism that influences developmental pathways and competitive opportunities. A related ethical question however emerges: should sports practitioners be guided, or even encouraged to target specific events and athletes to maximise the likelihood of international medal success? This mirrors ethical debates already present in the literature around tailoring national Para sport policies to improve competitive outcomes at an international level (40).

A 2012 opinion piece entitled *Why Talent Needs Trauma* (46)*,* argued that athletes who progress through talent pathways often share a history of significant adversity, including major life challenges. Given the distinctions between acquired and congenital impairments discussed above, it may be worthwhile exploring the role of perceived trauma in the Para athlete population. Future research could investigate whether a relationship exists between trauma, understood broadly as adverse life experiences such as sustaining an injury or facing social exclusion as a result of living with a congenital impairment, and sporting success among Para athletes.

Building on these impairment-related considerations, the RAE appears to be shaped by a several interconnected factors, including broader social structures, economic conditions, and specific impairment characteristics. Previous Para sport research has highlighted meaningful developmental distinctions between athletes with acquired vs. congenital impairments (9, 25, 47). A direct comparison of the RAE across these groups represents an important avenue for future inquiry. When considered alongside emerging evidence on athlete well-being (48) and predictors of medal performance (38), such comparative analyses may yield a more refined understanding of how impairment-related factors contribute to individual athlete trajectories and competitive outcomes. Collectively, these insights highlight the need for coaches and other TIdD stakeholders in Para sport to attend closely to the RAE and the maturation–selection hypothesis, thereby helping to minimise bias in selection processes and in wider TIdD practices.

The use of non-disabled athletes as comparison groups has been utilised in some studies (32, 33). Athletes with cerebral palsy (CP) often exhibit spasticity, asymmetry, and reduced neuromuscular control, which can hinder maximal force production and consistency in sprint mechanics (49, 50). As such, direct comparison with non-disabled athletes may be inappropriate unless the intention is to explicitly highlight differences in movement capabilities. Beckman et al. (33) and Connick et al. (32) both investigated physical tests in the context of classification. Measures used for classification are required to be resistant to the effects of training (13). Accordingly, these studies compared athletes with impairments to non-disabled athletes, as improvements in strength or conditioning should not significantly influence the outcomes of such tests.

In contrast to athletes with CP, athletes with VI typically retain neuromuscular function, allowing for more consistent power production (51), which may provide reason for the inclusion of athletes with VI only by Loturco et al., with no comparisons to athletes without VI. The contrast between these two papers could be reflective of the treatment of different classifications within the Para sporting system: those athletes who are considered “less impaired”, or whose physical function is more aligned with that of athletes without impairments may experience priority in terms of TD over those athletes who have more severe impairments. This prioritisation could manifest as athletes with less severe impairments progressing through talent pathways faster and thus receiving greater access to resources.

Contrasting other sprint performance research, Andrews et al. (30) investigated the usefulness of kinematics norms for Paralympic sprinters through descriptive analysis. The findings of this study suggest that the comparison of kinematics between Para athlete and non-Para norms may help coaches identify key focus areas for enhancing sprint starts, especially if the athlete uses starting blocks. Of note, Andrews et al., highlighted unique findings for each impairment included in their participant group. This highlights the need for classification-specific benchmarking within Para sport, as reliance on non-Para norms may not adequately reflect the functional constraints and performance determinants of different impairment groups. Developing classification-sensitive testing frameworks may therefore support more accurate talent identification and more effective alignment between assessment practices and athlete development pathways.

### 4.1 Limitations

Although this scoping review provides an important overview of the existing literature on TIdD in PA, limitations inherent to the scoping review methodology should be acknowledged. TIdD in Para sport is in its infancy, and therefore methods of TIdD in PA are limited, which may contribute to a lack of standardised processes in applied sporting contexts. This is particularly relevant in this review, as research designs vary widely and sample sizes are varied due to the diverse and relatively fragmented athlete population.

The broad nature of scoping reviews can lead to a more superficial analysis and the inclusion of studies which may only have moderate relevance to the research question. For example, this review includes studies related to Para sport rather than PA specific research only. While we identified key themes (e.g., diversity and classification-related factors), these concepts were not explored in depth across impairment types or classification groups. We further acknowledge the context specificity of the studies in this review and note that cross comparison is limited due to the various sporting nations, and thus related factors such as demographics, national funding models, and athlete availability. This may oversimplify the complex biopsychosocial nature of Para sport and dilute the findings specific to PA.

## 5 Conclusion

This scoping review provides a comprehensive overview of the current landscape of TIdD practices in PA. The findings reveal a limited but gradually expanding body of literature, with most research published in the past decade, underscoring the relative newness and under-explored nature of this field. While the included studies span a range of Para sporting nations and research designs, significant gaps remain, particularly regarding research focused on female athletes, impairment-specific investigations, and research related to field events and the newly included Paralympic event of frame running. The predominance of studies involving male participants and the lack of female-only research highlight the need for more inclusive and representative investigations.

Moreover, the review demonstrates that existing knowledge in TIdD is adapted from non-disabled sport, often without sufficient consideration of the unique complexities inherent to PA. This “copy and paste” approach may not adequately address the specific needs of Para athletes, suggesting that bespoke models and frameworks are required for effective TD in this context. While this review demonstrates the current TIdD research in PA, it may also reproduce the structural biases of the Para sport system.

The included studies highlight the usefulness of practical physical assessments, including strength, co-ordination, ROM, kinematic analyses, and assessment of anthropometric data, for the purpose of identifying and classifying individuals who may possess athletic talent. Outside of physical testing, other methods of identifying athletes who may have an increased likelihood of success in PA include examining demographic data, such as birthdates and classification related factors.

Overall, this review highlights the urgent need for further empirical research, especially studies that address gender disparities and the diverse range of impairments present in PA. Across all studies, classification categories were reported as contextual factors influencing athlete experiences and abilities; however, these were rarely examined in relation to broader system structures such as pathway accessibility or event availability. Consequentially, we conclude that research in PA is both about classification and structured by it, which reinforces its dual role as a performance determinant and a research boundary. Future research should prioritise longitudinal and classification-specific investigations to better inform evidence-based policies and practices, ultimately supporting the growth and success of PA athletes worldwide. To advance athlete equality, system level reform in classification governance, pathway design and resource distribution must parallel scientific progress.
