# Supplementary material for: From potential to podium: what we still don't know about talent identification and development in Para athletics, a scoping review
Source: Front Sports Act Living. 2026 Jun 15;8:1782132. doi: 10.3389/fspor.2026.1782132 (PMC13311012; doi:10.3389/fspor.2026.1782132)
Supplement: Supplementary file 1 [file Supplementaryfile1.pdf]

Supplementary File 1. Search Terms

Para Athletics OR Paraathletics OR Parathletics OR Para-Athletics OR Paralympic Athletics OR Paralethics OR Para sport OR Para-Sport OR Para-running OR Para running OR Para Track and Field OR Para Track OR Para-Track OR Para Field OR Para-Field OR Para throwing OR Para jumps OR Paralympics OR Para olympics OR Wheelchair sport OR Wheelchair racing
AND
Athlete OR Runner OR Thrower OR Jumper OR Coach OR Support staff OR Scout OR Stakeholders
AND
Talent identification OR Talent ID OR Talent development OR Athletic development OR Athlete development OR Athlete progression OR Athlete pathway OR Athletic pathway OR Talent academy OR Development academy OR Talent acquisition OR Sport acquisition OR Athlete progression OR Athletic progression OR Identification OR Development
