# Supplementary material for: From potential to podium: what we still don't know about talent identification and development in Para athletics, a scoping review
Source: Front Sports Act Living. 2026 Jun 15;8:1782132. doi: 10.3389/fspor.2026.1782132 (PMC13311012; doi:10.3389/fspor.2026.1782132)
Supplement: Supplementary file 2 [file Supplementaryfile2.pdf]

Supplementary File 2. Inclusion and Exclusion Criteria

	**Inclusion criteria**	**Exclusion criteria**
**Population(s)** *types/characteristics of participants; ages of participants; health conditions of participants; etc.*	Para athletics<br>Any age<br>Athletes with an eligible impairment<br>Both genders	Non-disabled sports<br>Another identified Para sport* (outside of athletics)<br>Athletes who do not have an eligible impairment*<br>Coaches/ scouts involved in non-disabled sports<br>Special Olympics<br>Olympics
**Concept(s)**<br>*Are there any interventions or activities that are of interest?*	Talent identification assessments<br>Talent development testing (repeated tests)<br>Training interventions (in the context of developing Para athletes)<br>Identification of variables associated with sporting success	Athlete development research in non-disabled sport<br>Para sport articles in contexts outside of talent identification/ development (e.g., Para athletes' perception on disability sport activism; advancements in the technology for Para sport equipment; injury risk in Para sport; doping; Para sport laws/ rules)<br>Articles on disability sport outside the context of TIdD<br>Exercise interventions with people with disabilities
**Context(s)**<br>*In what setting is your concept relevant?*	N/A	N/A

**Date or language criteria**	Any language	N/A

*The IPC reintroduced intellectual impairments as an eligible impairment in Para athletics from the London Paralympic Games in 2012, allowing intellectually impaired athletes to be classified by their intellectual impairment in track and field events. Therefore, studies on the topic of intellectual impairments in athletics prior to 2012 were excluded.
