# Supplementary material for: From potential to podium: what we still don't know about talent identification and development in Para athletics, a scoping review
Source: Front Sports Act Living. 2026 Jun 15;8:1782132. doi: 10.3389/fspor.2026.1782132 (PMC13311012; doi:10.3389/fspor.2026.1782132)
Supplement: Supplementary file 3 [file Supplementaryfile3.pdf]

Supplementary File 3. Quality Appraisal Assessment

**Authors**	**A clear description of the inclusion and exclusion criteria was provided**	**The trials were randomized**	**The method used to generate the random allocation sequence was described**	**Sample size was justified**	**Attempts were made to control and/or monitor pre-trial condition**	**Design incorporated measures of important baseline variable**	**Subjects were blinded**	**Investigators were blinded**	**Methods and successfulness of blinding were describe**	**details were provided regarding the inability of a subject to complete study requirement**	**Statistical methods described**	**Primary outcome measurement and variability reported**	**Results of statistical comparisons reported**	**Methods used to assess adverse effects described**	**Reproducibility of the primary outcome measure (s) was reported**	**A familiarization of the performance test was conducted**	**Total score (%)**
**Andrews BS; et al, 2011**	1	NA	NA	NA	NA	NA	NA	NA	NA	0	1	1	1	0	1	1	75%
**Loturco I, et al. 2015**	1	NA	NA	0	NA	NA	NA	NA	NA	0	1	1	1	0	1	1	67%
**Connick MJ; et al, 2015**	1	NA	NA	0	NA	NA	NA	NA	NA	1	1	1	1	1	1	1	89%
**Beckman EM; et al, 2016**	1	NA	NA	0	NA	NA	NA	NA	NA	0	1	1	1	1	1	1	78%
**Bayarslan B; et al, 2023**	1	NA	NA	1	NA	NA	NA	NA	NA	NA	1	1	NA	NA	0	NA	80%
**Teymouri M; et al, 2023**	1	NA	NA	0	NA	NA	NA	NA	NA	0	1	1	1	NA	1	NA	71%
**Peake R; Davies LE, 2024**	1	NA	NA	1	NA	NA	NA	NA	NA	1	1	1	NA	0	1	0	75%
**Solon-Júnior LJ; et al, 2024**	1	NA	NA	1	NA	NA	NA	NA	NA	NA	1	1	1	NA	1	NA	100%
**Severin AC; et al, 2025**	1	NA	NA	0	NA	NA	NA	NA	NA	NA	1	1	1	NA	1	NA	83%
